# Bah humbug! Association between sending Christmas cards to trial participants and trial retention: randomised study within a trial conducted simultaneously across eight host trials

**DOI:** 10.1136/bmj-2021-067742

**Published:** 2021-12-14

**Authors:** Elizabeth Coleman, Catherine Arundel, Laura Clark, Laura Doherty, Katie Gillies, Catherine Hewitt, Karen Innes, Adwoa Parker, David Torgerson, Shaun Treweek

**Affiliations:** 1 York Trials Unit, Department of Health Sciences, University of York, York, UK; 2 Health Services Research Unit, University of Aberdeen, Aberdeen, UK

## Abstract

**Objectives:**

To determine the effectiveness of sending Christmas cards to participants in randomised controlled trials to increase retention rate at follow-ups, and to explore the feasibility of doing a study within a trial (SWAT) across multiple host trials simultaneously.

**Design:**

Randomised SWAT conducted simultaneously across eight host trials.

**Setting:**

Eight randomised controlled trials researching various areas including surgery and smoking cessation.

**Participants:**

3223 trial participants who were still due at least one follow-up from their host randomised controlled trial.

**Intervention:**

Participants were randomised (1:1, separately by each host trial) to either received a Christmas card in mid-December 2019 or to not receive a card.

**Main outcome measure:**

Proportion of participants completing their next follow-up (retention rate) within their host randomised controlled trial.

**Results:**

1469 participants (age 16-94 years; 70% (n=1033) female; 96% (813/847) white ethnicity) across the eight host randomised controlled trials were involved in the analysis (cut short owing to covid-19). No evidence was found of a difference in retention rate between the two arms for any of the host trials when analysed separately or when the results were combined (85.3% (639/749) for cards versus 85.4% (615/720) for no card; odds ratio 0.96, 95% confidence interval 0.71 to 1.29; P=0.77). No difference was observed when comparing just participants who were due a follow-up in the 30 days after receiving the card (odds ratio 0.96, 0.42 to 2.21). No evidence of a difference in time to complete the questionnaire was found (hazard ratio 1.01, 95% confidence interval 0.91 to 1.13; P=0.80). These results were robust to post hoc sensitivity analyses. The cost of this intervention was £0.76 (€0.91; $1.02) per participant, and it will have a carbon footprint of approximately 140 g CO_2_ equivalent per card. One benefit of this approach was the need to only submit one ethics application.

**Conclusions:**

Sending Christmas cards to participants in randomised controlled trials does not increase retention. Undertaking a SWAT within multiple randomised controlled trials at the same time is, however, possible. This approach should be used more often to build an evidence base to support selection of recruitment and retention strategies. Although no evidence of a boost to retention was found, embedding a SWAT in multiple host trials simultaneously has been shown to be possible.

**Study registration:**

SWAT repository https://www.qub.ac.uk/sites/TheNorthernIrelandNetworkforTrialsMethodologyResearch/FileStore/Filetoupload,846275,en.pdf#search=SWAT%2082.

**Figure:**
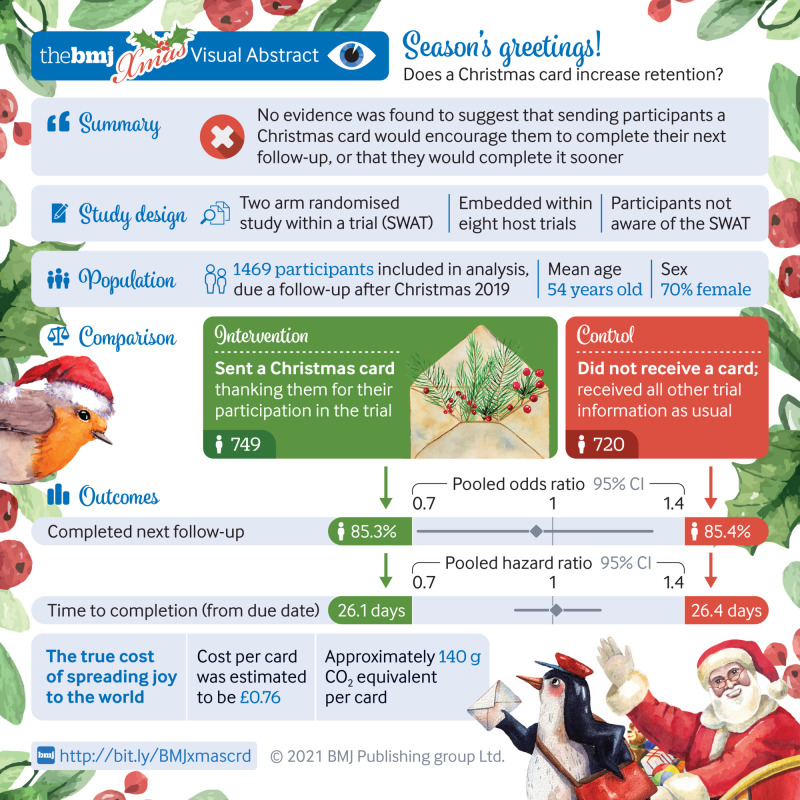


## Introduction

The Charles Dickens classic *A Christmas Carol*, never out of print since publication in 1843, describes how Scrooge is visited by ghosts of Christmas past, present, and future. The Ghost of Christmas Past explains that he wears a chain he forged in life, stating: “I made it link by link, and yard by yard; I girded it on of my own free will, and of my own free will I wore it.”

This ghost is not alone in carrying a chain. Trials too haul chains, often wearily. A few links for an unclear question, one more for a poorly worded information leaflet, another for the consent form, and too many to count for the outcomes. Link by link, trials wear the chains we forge for them in design. One consequence of this chain may be that participants find the demands of the trial too taxing and stop responding. This leaves us with poor retention—in the same way that old Ebenezer, with his miserly ways and lack of Christmas cheer, was left him a staff turnover problem. Poor retention is vexing because it can adversely affect study validity.^1^ ^2^ ^3^

To mitigate this problem, researchers implement strategies to try to improve retention, such as sending text messages or Christmas cards.^1^ ^3^ ^4^ Although Scrooge is unlikely to have sent Christmas cards, many trial teams do. A 2017 survey of UK registered clinical trials units found that 40% had previously used Christmas cards as a retention strategy despite a lack of evidence supporting their use.^4^ The implementation of this strategy requires both money (for printing and posting) and staff time. This expense may be viewed as justifiable if evidence shows an increase in retention rates; otherwise it contributes to research waste, and trial teams should implement a strategy shown to work.

However, many, if not most, retention strategies have little robust evidence to guide their use. In the absence of high certainty evidence, one way of changing this situation would be to evaluate the effectiveness of retention strategies by embedding a study within a trial (SWAT) into the host trial, whereby participants are randomised to receive different strategies and the effect on retention is measured.^5^

As single SWAT evaluations are not usually powered to show a small difference in effectiveness, owing to their limited size, many replications of a SWAT are needed in different settings and populations to allow for a fair evaluation of effectiveness to be made.^6^ Thus, years may be needed for enough SWAT replications to be done to reach a conclusion on the intervention’s effectiveness. One way to speed up this process, and allow for rapid collection of evidence, would be to plan to do the same SWAT simultaneously in several host trials. The feasibility of conducting simultaneous SWATs of the same intervention has been investigated only a few times.^7^ ^8^ This frugal, evidence based approach to retention strategy selection is one that Scrooge, we are sure, would approve of. Therefore, the aim of this study was to run a SWAT to evaluate the sending of Christmas cards as a trial retention strategy, across multiple host trials simultaneously.

## Methods

### Design

This SWAT was registered with the Northern Ireland Hub for Trial Methodology Research SWAT Repository (SWAT 82), and each of the host trials was registered individually (table 1).^9^ The two arm study was embedded within eight host UK randomised controlled trials simultaneously in December 2019, by two clinical trials units: York Trials Unit (YTU) and Aberdeen’s Centre for Healthcare Randomised Trials (CHaRT). We invited all host trials at the two coordinating units that were anticipated to be following up participants after Christmas 2019 to participate, and we assessed those wanting to participate for their suitability for this intervention (that is, in terms of trial population and follow-up method). Trials could continue to use any other retention strategies that were planned, as a benefit from these would be equally applicable to both arms of this SWAT. The eight host trials included in the evaluation (C-Gall, CPIT-3, DISC, FUTURE, ProFHER-2, PUrE, REFLECT, and SWHSI-2) span a wide range of research areas, including dental hygiene, surgery, and smoking cessation in pregnant women (table 1).^10^ ^11^ ^12^ ^13^ ^14^ ^15^ ^16^ ^17^ We planned a follow-up period of one year, to allow for each participant in the SWAT to have had at least one follow-up.

**Table 1:** Descriptions of host trials

Acronym (trials unit)	Title/description (registration)	Interventions	Area	Primary outcome	Follow-up method	Target sample size
**C-Gall** 10 ** (CHaRT)**	A randomised controlled trial comparing laparoscopic cholecystectomy with observation/conservative management for preventing recurrent symptoms and complications in adults with uncomplicated symptomatic gallstones (ISRCTN55215960)	Laparoscopic cholecystectomy or conservative management.	Surgery: gallbladder	Short Form-36 at 18 months	Postal	430
**CPIT-3** 11 ** (YTU)**	The smoking cessation in pregnancy incentives trial: A multi-centre phase 3 randomised controlled trial (ISRCTN15236311)	Both groups receive smoking cessation service support and contingent shopping vouchers. Intervention group receive additional shopping vouchers (up to £400)	Smoking in pregnancy	Self-reported abstinence from smoking for 8 weeks	Telephone	940
**DISC** 12 ** (YTU)**	A pragmatic multi-centre randomised controlled non-inferiority, cost effectiveness trial comparing injections of a collagenase into the cord to surgical correction in treatment of moderate Dupuytren’s contracture in adult patients (ISRCTN18254597)	Injection of collagenase or surgery	Surgery/drug: hand	Patient evaluation measure at 1 year	Clinic/postal	710
**FUTURE** 13 ** (CHaRT)**	A superiority randomised clinical trial to evaluate the effectiveness and cost effectiveness of invasive urodynamic investigations in management of women with refractory bladder symptoms (ISRCTN63268739)	Urodynamics plus comprehensive clinical assessment or comprehensive clinical assessment only	Female bladder weakness	Patient Global Impression of Improvement at 15 months	Postal	1096
**ProFHER-2** 14 ** (YTU)**	A three-arm randomised controlled trial to assess the effectiveness and cost-effectiveness of reverse shoulder arthroplasty verses hemi-arthroplasty verses non-surgical care for acute three- and four-part fractures of the proximal humerus in patients over 65 (ISRCTN50850043)	Reverse shoulder arthroplasty or hemi-arthroplasty or non-surgical	Surgery: shoulder	Oxford Shoulder Score at 2 years	Clinic/postal	380
**PUrE** 14 ** (CHaRT)**	The clinical and cost effectiveness of surgical interventions for stones in the lower pole of the kidney (ISRCTN98970319)	Extracorporeal shockwave lithotripsy or percutaneous nephrolithotomy or flexible ureterorenoscopy with laser lithotripsy	Surgery: kidney	EQ-5D-5L at 12 weeks	Postal	1044
**REFLECT** 15 ** (CHaRT)**	A randomised controlled trial to evaluate the effectiveness and cost benefit of prescribing high dose fluoride toothpaste in preventing and treating dental caries in high-risk older adults (ISRCTN11992428)	Prescription of 5000 ppm fluoride toothpaste or usual care	Dental	Proportion of participants receiving dental care due to caries at 36 months	Postal	1174
**SWHSI-2** 17 ** (YTU)**	A pragmatic multicentre randomised controlled trial to assess the clinical and cost effectiveness of negative pressure wound therapy versus usual care for surgical wound healing by secondary intention (ISRCTN26277546)	Negative pressure wound therapy or usual care (normal dressing)	Wound healing	Time (days) to wound healing	Postal	696

CHaRT=Centre for Healthcare Randomised Trials (Aberdeen); YTU=York Trials Unit.

#### Protocol changes

Owing to the covid-19 pandemic, many trials had to alter the way in which they followed up their participants, including implementation of other retention strategies or switching from in-person follow-up to remote data collection, which could introduce more heterogeneity. Additionally, how participants’ retention behaviour may change because of the pandemic and what the future was for many of the trials were unknown. We (all authors of this paper) discussed this and decided that, to allow for a true evaluation of this intervention, we would stop the SWAT early and include only follow-ups that were due on or before 31 March 2020 rather than mid-December 2020 (that is, to evaluate the effect on outcome data collection up to three months rather than the originally planned 12 months).

### Participants and randomisation

This SWAT’s sample size was constrained by that of its host trials; therefore, we did no formal power calculation, as is standard practice for SWAT evaluations.^2^ ^5^ In this evaluation, as the SWAT was done simultaneously in multiple host trials, the sample size was larger than is usual in a single SWAT.

We implemented this SWAT within all eight trials at the same time (December 2019), and any participants of the host trials who were still in follow-up, regardless of what time point they were at, were eligible to be included in the SWAT, as long as they had a postal address recorded. We gave some consideration as to whether inclusion of specific participants was appropriate, particularly in the pregnancy trial if a known negative outcome had occurred, and inclusion of participants was at the discretion of each of the individual trial teams.

As we and our ethics committee considered the SWAT to be low risk, and as all participants in the SWAT were to be blinded to their participation, we obtained no further consent from participants. Additionally, each host trial had consent from their participants to contact them regarding research, to use their data for research purposes, and to share their anonymised data for research. The host trials collected all data routinely, according to their protocols.

Randomisation was done separately for each host trial. The host trials being run by YTU each used randomisation which was stratified by host trial allocation, using blocks of varying size (all used four and six, and all bar SWHSI-2 also used two), to ensure balance as some of the trials to be included had a low sample size at the point of randomisation. Trials from CHaRT used simple randomisation. All schedules used a 1:1 ratio, and the randomisation was done by a person not involved in the preparing and sending of the cards.

### Intervention

**Fig 1:**
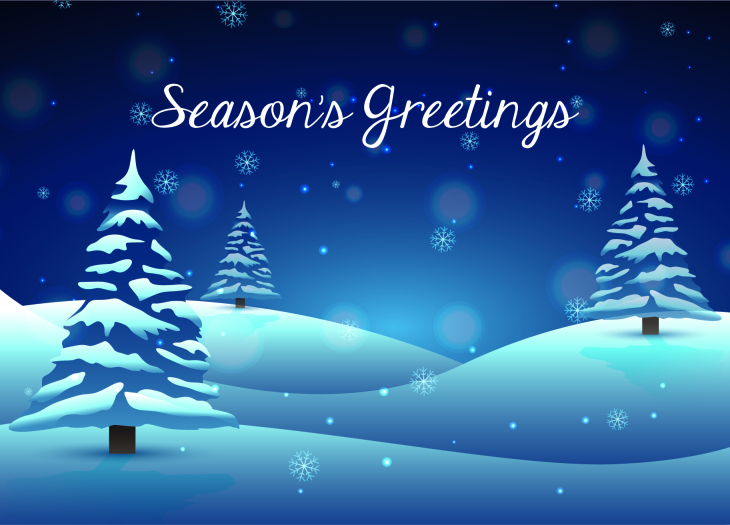
Front of Christmas card

The intervention to be tested within this SWAT evaluation was the sending of a Christmas card to participants in the host trials. The card selected featured a snowy winter scene and had the words “Season’s Greetings” on the front (fig 1). All of the cards contained the same message: “Thank you for taking part in [TRIAL ACRONYM]. Wishing you a Merry Christmas and a Happy New Year. From the [TRIAL ACRONYM] Study Group.” However, this was tailored slightly to the specific host trial, by including the signature from the chief investigator, or local principal investigator for CPIT-3, the signatures of the trial office team for the CHaRT trials, and the study logo. The respective trial teams put the Christmas cards into envelopes while listening to Christmas classics (Mariah Carey is an Aberdeen favourite), ate mince pies, and generally had a jolly time. Each host trial posted cards to participants allocated to the SWAT intervention between 11 and 17 December 2019, depending on the host trial, as second class letters by Mailmark franking via Royal Mail, expected to arrive in two to three working days. The control group received no card, but all other follow-up processes remained as per the host trial protocol.

### Outcomes

The primary outcome for this evaluation was the proportion of participants who completed their next follow-up (retention rate); we measured this as the number of completed follow-ups divided by the number of follow-ups that were due. When a participant had multiple follow-ups within the SWAT follow-up period, we included only the first one. Secondary outcomes were time to complete the follow-up (defined as number of days between follow-up due and follow-up complete), cost per card sent (including staff time and printing and postage costs), and cost per additional participant retained (if applicable)

We also explored the effect of the Christmas card on the subgroup of participants who were due to receive a follow-up shortly (up to 30 days) after the expected delivery date of the card. We theorised that if this intervention influenced retention, the effect would be most prevalent in participants who were due to have their follow-up shortly after receipt of the card, as those whose follow-up was due later may not remember receiving the card.

We also included a post hoc analysis to calculate CO_2_ emissions of this Christmas card SWAT. Additionally, we ran post hoc sensitivity analyses to explore the effect of the baseline imbalance of sex on the primary outcome and time to completion for only postal follow-ups, as a quicker response is applicable only in these follow-ups.

### Statistical analysis

We used Stata v.16 for all analyses, using the principles of intention to treat, whereby we analysed all participants according to the group to which they were randomised, regardless of whether they received the intervention. We used a 5% significance level and excluded participants from the analysis if they had been randomised in error or had withdrawn from follow-up before the posting of the card but had been randomised into the SWAT.

All analysis models were adjusted for the host trial allocation (that is, their control/intervention allocation from the respective main trial) and the SWAT allocation and were run for each host trial separately. No adjustment was made for baseline imbalance in the main analyses. We used a random effects meta-analysis to combine the results of the individual trials. The primary analysis used a logistic regression model to compare the retention rate between the two arms. We analysed time to completion (days between follow-up due and follow-up complete) by using a Cox proportional hazards regression; participants who completed their follow-up on time or early had their time set to 0.1, those who did not complete it or completed it more than 90 days after the due date were censored at 90 days, and those who withdrew after their follow-up was due were censored at their withdrawal date. We used Schoenfeld residuals to assess the assumptions for this model.^18^

We did a subgroup analysis by rerunning the primary analysis model including only those participants whose follow-up was due within 30 days of the expected delivery date for the Christmas card for that trial. We did a sensitivity analysis in which the primary analysis model was rerun for each host trial with further adjustment for sex; we then also combined these results in a meta-analysis. In a second sensitivity analysis, the time to completion analysis was rerun including only participant follow-ups done by post (that is, excluding CPIT-3 completely and any clinic follow-ups from ProFHER-2 and DISC).

We present the results for each model fitted, with the reference group being participants in the intervention arm. We present the results according to CONSORT guidelines for randomised controlled trials, with appropriate adjustments where these do not apply to SWATs.

We calculated the average cost per card sent as the sum of the cost per card for preparation, postage, and printing of the card. As different numbers of cards were involved at each of the three stages, the cost per card for each element was calculated separately, to give the most accurate estimate for the respective element. We calculated the cost per card as the total of the cost for each element divided by the number of cards involved. We calculated this as an overall cost for the SWAT, rather than for each host trial, to give a better estimate of the true cost. We recorded staff time and grade for each member of staff involved in packaging the Christmas cards; we used the salary of the midpoint of the grade band for each member of staff, from their respective university (figures were obtained in November 2020). We calculated the associated cost for each member of staff by using the time they had spent preparing the cards (in hours) multiplied by their associated hourly pay. We set the cost of postage per card as the cost for second class Royal Mail using Mailmark franking in December 2019 and recorded the cost of printing from the printing order. We then summed these costs per card to provide a total cost per card. Should we find that the intervention was effective, we would divide the total cost by the number of additional participants retained, to give a cost per additional participant retained.

We based assumptions for the CO_2_ emissions calculation on previous research, assuming that the card weighs 10 g, is printed on recycled paper, and is posted and recycled in the UK.^19^ We included only cards printed and sent in this analysis.

### Patient and public involvement

CHaRT has previously sent Christmas cards to trial participants, but not as part of an evaluation. We consulted a patient and public involvement (PPI) group consisting of six members from the Health Services Research Unit PPI Partnership on the design and content of the card in November 2019. The group reviewed two different card designs and the wording inside the card and were offered the chance to provide feedback on any other aspect. Most of the group members agreed on the card that was ultimately used in all host trials (fig 1) and deemed the card to be acceptable.

## Results

A total of 3223 participants were randomised to be included in this SWAT—1617 to receive the Christmas card and 1606 to not receive a card (table 2). We excluded three participants who had no contact address recorded and so were not eligible to be included in the SWAT. Additionally, one participant was randomised three times in error; we included the first allocation in the analysis and excluded the other two allocations (one in each arm). At least one card was returned as the participant no longer lived at the stated address; however, they are included in the analysis under the intention to treat principle.

**Table 2:** Baseline characteristics of participants included in primary analysis (due for follow-up on/before 31 March 2020*)*, by arm, for each host trial and overall. Values are numbers (percentages) unless stated otherwise

Trial	Intervention—participants allocated to receive card						Control—participants allocated not to receive card				
	Randomised*	Analysed	Mean (SD; range) age, years	Female sex	White ethnicity		Randomised*	Analysed	Mean (SD; range) age, years	Female sex	White ethnicity
C-Gall	205	110 (54)	50.3 (15.1; 21-80)	86 (78)	96 (87)		197	108 (55)	48.5 (14.8; 19-80)	86 (80)	92 (85)
CPIT-3	242	157 (65)	28.4 (5.7; 16.8-43.0)	157 (100)	155 (99)		242	155 (64)	27.4 (5.7; 16.8-41.9)	155 (100)	155 (100)
DISC	205	125 (61)	67.5 (8.7; 31.4-84.8)	22 (18)	125 (100)		203	117 (58)	67.7 (8.5; 39.2-89.2)	28 (24)	117 (100)
FUTURE	273	155 (57)	60.8 (12.8; 23.1-82.3)	155 (100)	-		270	138 (51)	63.3 (13.2; 25.3-94.4)	138 (100)	-
ProFHER-2	45	17 (38)	77.4 (6.8; 65.5-87.6)	13 (76)	17 (100)		47	15 (33)	75.2 (5.2; 68.8-87.0)	13 (87)	13 (87)
PurE	75	20 (27)	58.2 (14.4; 29.3-82.1)	8 (40)	-		74	27 (36)	53.1 (12.5; 27.9-75.8)	14 (52)	-
REFLECT	546	144 (26)	65.0 (8.5; 50.1-85.7)	78 (54)	-		547	138 (25)	64.0 (8.7; 50.6-87.8)	70 (51)	-
SWHSI-2	26	21 (81)	61.3 (7.8; 49.2-75.6)	3 (14)	21 (100)		26	22 (85)	59.5 (13.3; 30.7-80.1)	7 (32)	22 (100)
Overall	1617	749 (46.3)	54.7 (18.0; 16.8-87.6)	522 (69.7)	414/430 (96)		1606	720 (44.8)	53.9 (18.5; 16.8-94.4)	511 (71.0)	399/417 (96)

* Includes those randomised in error.

**Fig 2:**
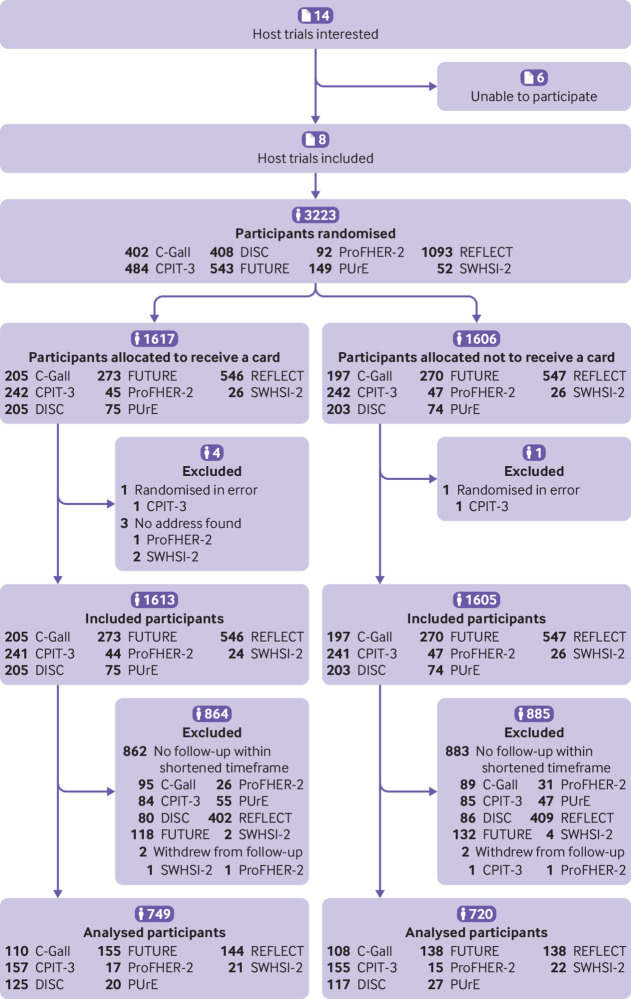
Flow of participants through study within a trial

Only 1469 (749 randomised to receive the card; 720 not) participants were due a follow-up on or before 31 March 2020 and thus are included in the analysis (45.6% of 3223 randomised) (table 2). The percentage of randomised participants included in the analysis varied from 26% (282/1093, REFLECT) to 83% (43/52, SWHSI-2), as shown in table 2; this was due to both the frequency of the follow-ups and the stage the trial was at. For instance, the participants in SWHSI-2 were due their three month and six month follow-ups, whereas those in REFLECT were due their one year follow-up. Figure 2 shows the flow of participants through the SWAT. Table 2 shows the baseline characteristics of the participants included in the analysis. Most participants were female (1033/1469; 70%) and of white ethnicity (813/847; 96%). Additionally, as shown table 1, most of the trials implemented postal follow-up, which may not be typical of all trials.

### Primary outcome

The overall rate of completion of the next follow-up (retention rate), for all trials combined, was 85.4% (1254/1469 follow-ups completed); this ranged from 63% (27/43) to 96% (280/293) for overall completion per host trial (table 3). We observed similar levels of retention in the two arms overall—85.3% (639/749) for participants who received the Christmas card and 85.4% (615/720) for those who did not. The retention rate seen in most of these trials is in line with the median retention of 89% for a cohort of publicly funded UK trials as reported by Walter et al.^20^ The trials with lower retention rates (PUrE and SWHIS-2, as shown in table 3) have low sample sizes, so this may not reflect the retention of the trial once completed. We found no evidence of a difference in retention rate between the two arms for all eight of the host trials when analysed separately (table 3).

**Table 3:** Completion rates of follow-ups, details of primary outcome for each host trial, and combined meta-analysis results. Values are percentages (numbers completed/numbers due) unless stated otherwise

Trial	Sent a card	Not sent a card	Overall	Adjusted odds ratio (95% CI)*	P value
C-Gall	87 (96/110)	86 (93/108)	87 (189/218)	1.08 (0.49 to 2.37)	0.84
CPIT-3	90 (142/157)	90 (139/155)	90 (281/312)	1.09 (0.52 to 2.30)	0.81
DISC	84 (105/125)	87 (102/117)	86 (207/242)	0.77 (0.37 to 1.58)	0.47
FUTURE	95 (147/155)	96 (133/138)	96 (280/293)	0.66 (0.21 to 2.08)	0.48
ProFHER-2	82 (14/17)	80 (12/15)	81 (26/32)	1.05 (0.16 to 6.76)	0.96
PurE	65 (13/20)	70 (19/27)	68 (32/47)	0.88 (0.25 to 3.11)	0.84
REFLECT	75 (108/144)	75 (104/138)	75 (212/282)	0.97 (0.57 to 1.67)	0.92
SWHSI-2	67 (14/21)	59 (13/22)	63 (27/43)	1.38 (0.40 to 4.79)	0.61
Overall	85.3 (639/749)	85.4 (615/720)	85.4 (1254/1469)	0.96 (0.71 to 1.29)	0.77

* Reference is intervention arm.

**Fig 3:**
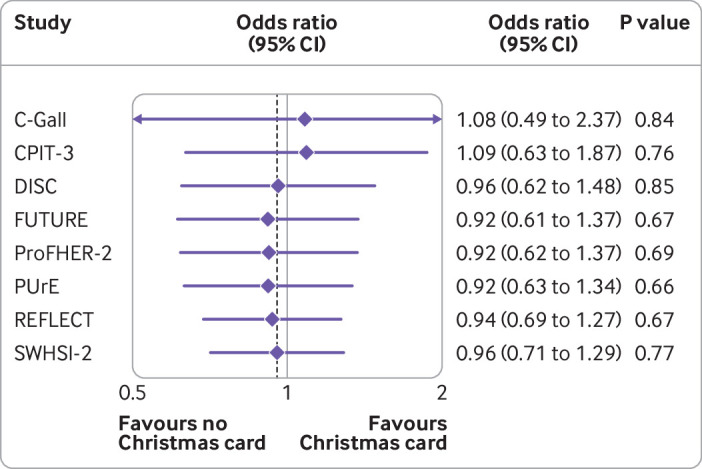
Cumulative meta-analysis of primary outcome (completion rate)

When we combined the results by using a random effects meta-analysis, we found no evidence of a difference in the retention rates between participants who received a Christmas card and those who did not (odds ratio 0.96, 95% confidence interval 0.71 to 1.29; P=0.77). Figure 3 shows a cumulative meta-analysis.

### Secondary outcomes

#### Follow-up due within 30 days

Only 20.8% (305/1469) of the participants in this SWAT evaluation were due a follow-up shortly after the delivery of the Christmas card. Of the 305 follow-ups included in this subgroup, 263 were completed—a retention rate of 86%, similar to that seen for all participants. This was similar between the two arms—85% for those who received a Christmas card (135/158) and 87% (128/147) for those who did not. Most of the trials had too few participants for the results of any analysis to be reliable, so we fitted a model only for CPIT-3 and DISC. FUTURE had enough participants for the model to be run, but all participants in the control group completed the follow-up, so an estimate cannot be obtained. Neither DISC nor CPIT-3 showed any evidence of a difference in retention rate between the two arms, considering just participants who were due a follow-up shortly after the delivery of the Christmas card (odds ratio 0.76, 0.24 to 2.39 (P=0.64) for DISC; 1.24, 0.37 to 4.23 (P=0.73) for CPIT-3). Combining these results in a meta-analysis gives an odds ratio of 0.96 (0.42 to 2.21). This result mirrors that found in the primary analysis; participants who were sent a card were less likely to complete their follow-up, although the result is not significant.

#### Time to complete

**Fig 4:**
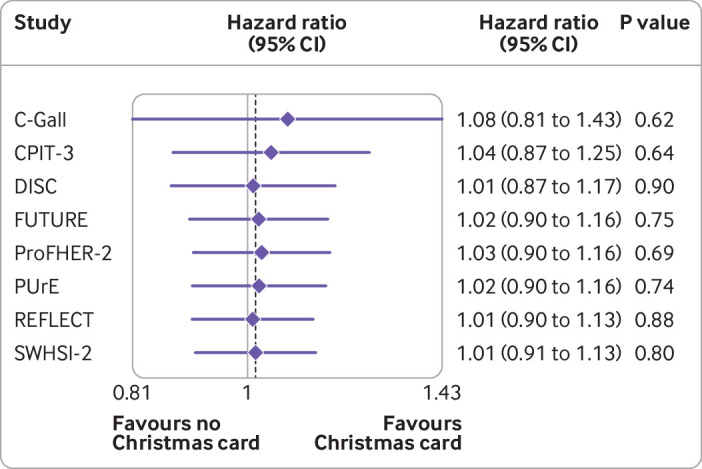
Cumulative meta-analysis of secondary outcome (time to completion)

On average, the follow-ups were completed 26.3 days after they were due; this was similar between the two arms (26.1 days for those who received a card and 26.4 for those who did not). The average time varied widely between the trials involved, most likely because of the method of follow-up (supplementary table A). For instance, CPIT-3 had no follow-ups that were completed early; the follow-up for this trial was done by a researcher telephoning a participant, so it could not be completed early. In some of the surgical trials, such as DISC, the follow-up may have been completed during a clinical follow-up appointment, which may have been scheduled early in a specified visit window for the participant’s convenience. For each of the host trials, we found no evidence of a difference in time to complete the follow-up between the two arms (supplementary table A), and the assumptions of proportional hazards held for each trial. The overall meta-analysis also supported the conclusion that no evidence exists of a difference between the two arms (hazard ratio 1.01, 95% confidence interval 0.91 to 1.13; P=0.80) (supplementary table A). Figure 4 shows a cumulative meta-analysis.

#### Cost per card

The costs below are detailed for the overall SWAT, including all Christmas cards that were sent (n=1617). A surplus of cards was ordered (n=1787), and owing to the way in which printing was done, an additional surplus was received and subsequently prepared in some instances. Preparing and packaging 1836 Christmas cards took 918 minutes (15.3 hours)—approximately 30 seconds per card. The members of staff involved with the preparation ranged from a grade 3 to a grade 7, with pay ranging from approximately £11.01 to £22.46 per hour. Each card was posted by second class Royal Mail using Mailmark franking at a cost of £0.41 each. Thus, the total cost of sending the Christmas cards was £1306.40 or £0.76 (€0.91; $1.02) per card sent. As we found no evidence of additional participants being retained, we did not calculate a cost per additional retained participant. Table 4 summarises the costs of sending the cards.

**Table 4:** Details of cost associated with Christmas cards

Task	Total cost (£)*	No of cards involved	Cost per card sent (£)*
Preparing cards	209.91	1836	0.11
Postage	662.97	1617	0.41
Printing and delivery	433.52	1787	0.24
Overall	1306.40	-	0.76

* £1 is equivalent to €1.19 and $1.34.

#### CO_2_ emissions

The estimate for the “carbon footprint” of a typical greetings card, such as a Christmas card, is 140 g CO_2_ equivalent.^19^ Based on the 1617 cards sent to participants in the intervention arm, the average amount of CO_2_ produced in this SWAT was 226 kg. This is equivalent to a return flight from Aberdeen to Leeds (York’s closest airport—approximately 230 miles direct distance). Based on previous research that identified 40% of clinical trials units having used Christmas cards as a retention strategy previously, and assuming that each UK clinical trials unit (n=53 UKCRC registered) would send cards in 10 host trials, each of moderate size (n=250), this would equate to a total CO_2_ emission of 4.5 tonnes per year.^4^ This does not account for additional trials that use Christmas cards but are conducted outside a registered clinical trials unit.

### Sensitivity analyses

Our sensitivity analyses looking at sex and postal-only time to completion confirmed our original conclusions. More details are in the supplementary materials.

## Discussion

In this study, we investigated the effect of sending a Christmas card to trial participants to boost retention. Although covid-19 shortened the follow-up period and limited the sample size, the data from more than 1400 participants across eight parallel evaluations show that sending a Christmas card to trial participants did not increase the retention rate, either in general or for participants who are due a follow-up within 30 days of receiving the card. Sending a Christmas card also did not influence the time taken for participants to complete the follow-up.

The intervention evaluated here may be one that trial teams favour, as it is relatively cheap to implement. However, other interventions exist that are cheap and show some benefit. For example, the effect estimate for including a pen with a trial questionnaire is a 2% (95% confidence interval 0% to 4%) increase in retention.^3^

Although we have concluded that sending Christmas cards does not result in a retention benefit for trials, whether it may have other benefits is worth considering. For instance, thanking/acknowledging participants’ time may be considered good manners, which may be worth the minor cost. However, this may be better suited to a “Thank you” card, which could offer an opportunity to provide text targeting things known to influence trial retention. Efforts to recruit and retain more diverse trial populations also point in the direction of alternative interventions with wide applicability and evidence of benefit. Finally, as sending Christmas cards is associated with a carbon cost, using scheduled opportunities to say thank you, such as questionnaire cover letters (see, for example, Goulao et al 21), may be wiser.

Overall, the retention rate in the eight host trials was in line with typical median retention rates of 89% seen in publicly funded UK randomised controlled trials.^20^ Perhaps a greater effect would be seen in trials with much lower retention of, say, 60%. Although we found no evidence of a difference in retention for this intervention, we acknowledge that any intervention that boosts retention is useful, as many small increases will accumulate and lead to a significant increase, which could ultimately influence the strength of the host trials’ findings.

This study showed that a single SWAT can be embedded successfully by multiple host trials across multiple clinical trials units simultaneously. This not only increases the speed at which evidence can be accumulated but has the additional benefit of allowing only one ethics application to be submitted to cover all the work, which will decrease the burden on individual trial teams.

### Strengths and limitations of study

When we initially planned this simultaneous SWAT, we hoped to involve more than 10 trials and more than 10 000 participants to ensure that the question could be answered by this one evaluation. The evaluation was delayed for various reasons, and some host trials could no longer embed the SWAT, which reduced the sample size. Additionally, although more than 3000 participants were randomised into this trial, the covid-19 pandemic meant that follow-up had to be cut short and as a result the sample size was reduced further. Moreover, we requested demographic data only for host trial participants due for follow-up by 31 March 2020 to reduce the burden on host trial teams during a difficult year. This means that we are unable to say whether our group of participants is different from those who would have been followed up after March 2020. Despite this, the lack of any clear evidence of benefit seen in our evaluation makes us confident that had the SWAT run for the entire year we would still have reached the same conclusion; if there was benefit to be had, it would be most apparent for participants being followed up close to Christmas. That effects get greater the further from Christmas follow-up occurs seems improbable.

This SWAT was successfully implemented across eight host trials, from two clinical trials units in the UK, at the same time. This is one of the first instances in which a SWAT evaluation has been undertaken in this way, and its success should influence other researchers to consider doing simultaneous SWATs in the future, to allow answers to the methodological questions that SWATs pose to be obtained more quickly.

### Future research

This trial was conducted in a wide variety of randomised controlled trials and associated participants, and no evidence of an effect was apparent. Thus, in line with Trial Forge Guidance 2,^6^ we consider that this question is answered, for UK adults at least, and recommend that future SWATs should prioritise the evaluation of other retention strategies, using a simultaneous design if possible.

However, consideration should be given to whether a future evaluation of this intervention in children and/or teenagers, or in those trials in which trial-participant relationships are more important, such as cancer trials, may lead to a different result. Additionally, this evaluation specifically focused on a Christmas card, which the PPI Group deemed appropriate for the populations in the associated host trials. Trial teams should consider evaluations of other cards, such as birthday or thank you cards, as well as exploring the effect of other culturally appropriate cards within different ethnic populations.

As we have concluded that this SWAT evaluation design is feasible and efficient, it should be used in future research. This design could be used to assess both recruitment and retention SWATs, as well as other methodological aspects such as protocol/treatment compliance strategies, and is suitable in most, if not all, research fields.

### Conclusion

“Bah humbug!” is the appropriate evidence based response to any colleague who suggests sending Christmas cards as a trial retention strategy to an adult population. Instead, trial teams should turn to strategies that have evidence of benefit, or where evidence is lacking trial teams should build SWATs into their trials to generate that evidence.

## What is already known on this topic

Poor retention is problematic in randomised controlled trials and can hamper the validity of trial results
Many retention strategies are used without evidence of their effectiveness
Evaluations of evidence based strategies are needed to ensure that the most effective strategies are being used to avoid research waste

## What this adds

In an adult UK population, Christmas cards are an ineffective retention strategy
Alternative retention methods should be used, and methods similar to this, such as birthday cards, may warrant an evaluation

## Data Availability

Data are subject to data sharing agreements and are not publicly available. Requests for data will be considered and should be sent to the corresponding author at izzy.coleman@york.ac.uk.

## Web extra

Extra material supplied by authors

Web appendix: Supplementary materials

## Notes

Contributors: EC prepared the initial draft of the manuscript and did the analysis. ST, KG, and KI prepared the protocol. KI assisted in the sharing of the data from the Aberdeen Centre for Healthcare Randomised Trials with the York Trials Unit for analysis. All authors contributed to revisions of the manuscript and approved the final version. EC is the guarantor. The corresponding author attests that all listed authors meet authorship criteria and that no others meeting the criteria were omitted.

The lead author (the manuscript’s guarantor) affirms that the manuscript is honest, accurate, and transparent account of the study being reported; that no important aspects of the study have been omitted; and that any discrepancies from the study as planned (and, if relevant, registered) have been explained.

Dissemination to participants and related patient and public communities: Despite the danger of being seen as the team that cancelled Christmas cheer in trials, we intend to disseminate these results widely through presentations at the PROMETHEUS hosted webinar series (https://www.york.ac.uk/healthsciences/research/trials/research/swats/prometheus/), via the international Trial Forge SWAT Network (https://www.trialforge.org/2021/06/swat_network/), and on the PROMETHEUS website (https://www.york.ac.uk/healthsciences/research/trials/research/swats/prometheus/). Additionally, the results will be presented at relevant conferences in the usual way and on social media in less usual ways.

Provenance and peer review: Not commissioned; externally peer reviewed.
