# Supplementary material for: Bah humbug! Association between sending Christmas cards to trial participants and trial retention: randomised study within a trial conducted simultaneously across eight host trials
Source: BMJ. 2021 Dec 14;375:e067742. doi: 10.1136/bmj-2021-067742 (PMC8669570; doi:10.1136/bmj-2021-067742)
Supplement: Supplementary file 1 — Web appendix: Supplementary materials [file cole067742.ww1.pdf]

Bah humbug! Association between sending Christmas cards to trial participants and trial retention: randomised study within a trial conducted simultaneously across eight host trials

Supplementary Material

Table A: Details on the proximity of the completion of the follow-up to when it was due, and results of the Cox Proportional Hazard model for all trials, and combined meta-analysis results

	Sent a card: Days between due and completion Mean (SD) (Min, Max)	Not sent a card: Days between due and completion Mean (SD) (Min, Max)	Overall: Days between due and completion Mean (SD) (Min, Max)	Hazards Ratio (95% CI)*	p-value
**C-Gall**	53.2 (40.9) (4, 156)	57.3 (46.0) (3, 158)	55.2 (43.4) (3, 158)	1.08 (0.81 to 1.43)	0.62
**CPIT-3**	10.1 (14.3) (0, 76)	10.5 (16.2) (0, 83)	10.3 (15.3) (0, 83)	1.02 (0.81 to 1.29)	0.85
**DISC**	12.5 (46.5) (-71, 335)	8.7 (22.2) (-42, 156)	10.7 (36.6) (-71, 335)	0.94 (0.71 to 1.23)	0.65
**FUTURE**	38.3 (44.3) (1, 188)	41.5 (39.9) (1, 172)	39.8 (42.2) (1, 188)	1.05 (0.83 to 1.33)	0.71
**ProFHER-2**	1.6 (13.9) (-29, 26)	7.3 (14.1) (-16, 28)	4.2 (14.0) (-29, 28)	1.25 (0.57 to 2.73)	0.57
**PurE**	14.7 (28.2) (-1, 103)	5.9 (9.7) (-1, 38)	9.5 (19.6) (-1, 103)	0.87 (0.43 to 1.79)	0.71
**REFLECT**	25.7 (25.5) (0, 132)	23.5 (23.7) (-8, 121)	24.6 (24.6) (-8, 132)	0.95 (0.73 to 1.25)	0.72
**SWHSI-2**	16.5 (18.7) (-3, 72)	30.5 (56.9) (4, 218)	23.2 (41.5) (-3, 218)	1.30 (0.61 to 2.77)	0.50
**Overall**	26.1 (38.5) (-71, 335)	26.4 (35.9) (-42, 218)	26.3 (37.2) (-71, 335)	1.01 (0.91 to 1.13)	0.80

*Reference arm is the intervention arm

Sensitivity analyses

Primary outcome further adjusted for gender

Due to an imbalance of gender in some of the host trials, a sensitivity analysis was undertaken. The results for each trial can be found in Table B. The results were robust and when combined in a meta-analysis produced an OR of 0.96, 95% CI 0.71 to 1.30, p = 0.78. The results suggest a negative impact on retention when sending a Christmas card, although these are non-significant.

Table B: Results of sensitivity analysis which further adjusted the primary analysis model for gender

	**Sent a card: % Complete (n complete /n due)**	**Not sent a card: % Complete (n complete /n due)**	**Overall: % Complete (n complete /n due)**	**Adjusted Odds Ratio (95% CI)***	**p-value**
**C-Gall**	87.3% (96/110)	86.1% (93/108)	86.7% (189/218)	1.09 (0.49 to 2.38)	0.84
**CPIT-3**	90.4% (142/157)	89.7% (139/155)	90.0% (281/312)	1.09 (0.52 to 2.30)	0.81
**DISC**	84.0% (105/125)	87.2% (102/117)	85.5% (207/242)	0.78 (0.38 to 1.61)	0.50
**FUTURE**	94.8% (147/155)	96.4% (133/138)	95.6% (280/293)	0.66 (0.21 to 2.08)	0.48
**ProFHER-2**	82.4% (14/17)	80.0% (12/15)	81.3% (26/32)	1.04 (0.16 to 6.75)	0.96
**PurE**	65.0% (13/20)	70.4% (19/27)	68.1% (32/47)	0.84 (0.23 to 3.02)	0.79
**REFLECT**	75.0% (108/14)	75.4% (104/138)	75.2% (212/282)	0.98 (0.57 to 1.69)	0.95
**SWHSI-2**	66.7% (14/21)	59.1% (13/22)	62.8% (27/43)	1.36 (0.38 to 4.84)	0.64
**Overall**	85.3% (639/749)	85.4 % (615/720)	85.4 % (1,254/1,469)	0.96 (0.71 to 1.30)	0.78

*Reference arm is the intervention arm

Time to completion: postal only

When repeating the time to completion analysis, with only those follow-ups that were done via postal questionnaire, the results were found to be similar to that when including all participants. This analysis affected three trials: CPIT-3, DISC and ProFHER-2. All participants from CPIT-3 were excluded. The full details can be found in Table C. A combined HR of 1.03 (95% CI 0.89 to 1.18, p=0.72) suggests a slight benefit from receiving the card, but the results are not statistically significant.

Table C: Results of sensitivity analysis where the time to completion analysis was repeated including only postal follow-ups.

	**Sent a card: Days between due and completion Mean (SD) (Min, Max)**	**Not sent a card: Days between due and completion Mean (SD) (Min, Max)**	**Overall: Days between due and completion Mean (SD) (Min, Max)**	**Hazards Ratio (95% CI)***	**p-value**
**C-Gall**	N=96<br>53.2 (40.9)<br>(4, 156)	N=93<br>57.3 (46.0)<br>(3, 158)	N=189<br>55.2 (43.4)<br>(3, 158)	1.08<br>(0.81 to 1.43)	0.62
**CPIT-3†**	N=0<br>-	N=0<br>-	N=0<br>-	-	-
**DISC†**	N=25<br>2.2 (4.9)<br>(-1, 17)	N=22<br>1.6 (6.0<br>(-1, 28)	N=47<br>2.0 (5.4)<br>(-1, 28)	0.91<br>(0.51 to 1.62)	0.76
**FUTURE**	N=147<br>38.3 (44.3)<br>(1, 188)	N=133<br>41.5 (39.9)<br>(1, 172)	N=280<br>39.8 (42.2)<br>(1, 188)	1.05<br>(0.83 to 1.33)	0.71
**ProFHER-2†**	N=8<br>4.3 (10.1)<br>(-7, 24)	N=5<br>11.4 (13.6)<br>(-1, 28)	N=13<br>7.0 (11.6)<br>(-7, 28)	1.73<br>(0.52 to 5.73)	0.37
**PurE**	N=13<br>14.7 (28.2)<br>(-1, 103)	N=19<br>5.9 (9.7)<br>(-1, 38)	N=32<br>9.5 (19.6)<br>(-1, 103)	0.87<br>(0.43 to 1.79)	0.71
**REFLECT**	N=108<br>25.7 (25.5)<br>(0, 132)	N=104<br>23.5 (23.7)<br>(-8, 121)	N=212<br>24.6 (24.6)<br>(-8, 132)	0.95<br>(0.73 to 1.25)	0.72
**SWHSI-2**	N=14<br>16.5 (18.7)<br>(-3, 72)	N=13<br>30.5 (56.9)<br>(4, 218)	N=27<br>23.2 (41.5)<br>(-3, 218)	1.30<br>(0.61 to 2.77)	0.50
**Overall**	N=411<br>34.1 (38.8)<br>(-7, 188)	N=389<br>35.7 (39.8)<br>(-8, 218)	N=800<br>34.9 (39.3)<br>(-8, 218)	1.03<br>(0.89 to 1.18)	0.72

*Reference arm is the intervention arm † some participants have been excluded from these trials as part of the sensitivity analysis
